# Data on optimization of the Karun-4 hydropower reservoir operation using evolutionary algorithms

**DOI:** 10.1016/j.dib.2019.105048

**Published:** 2020-01-08

**Authors:** Saeid Akbarifard, Mohammad Reza Sharifi, Kourosh Qaderi

**Affiliations:** a Candidate in Water Resources Engineering, Department of Hydrology and Water Resources, Faculty of Water Sciences Engineering, Shahid Chamran University of Ahvaz, Ahvaz, Iran; b Department of Hydrology and Water Resources, Faculty of Water Sciences Engineering, Shahid Chamran University of Ahvaz, Ahvaz, Iran; c Department of Water Engineering, Faculty of Agriculture, Shahid Bahonar University of Kerman, Kerman, Iran

**Keywords:** Optimization algorithms, Karun-4 reservoir, Hydropower operation, Moth swarm algorithm

## Abstract

This article describes the time series data for optimizing the hydropower operation of the Karun-4 reservoir located in Iran for a period of 106 months (from October 2010 to July 2019). The utilized time-series data included reservoir inflow, reservoir storage, evaporation from the reservoir, precipitation on the reservoir, and release of water through the power plant. In this data article, a model based on Moth Swarm Algorithm (MSA) was developed for the optimization of water resources. The analysis showed that the best solutions achieved by the MSA, Genetic Algorithm (GA), and Particle Swarm Optimization (PSO) were 0.147, 0.3026, and 0.1584, respectively. The analysis of these datasets revealed that the MSA algorithm was superior to GA and PSO algorithms in the optimal operation of the hydropower reservoir problem.

**Table:** Specifications Table

Subject	Water Resources Management
Specific subject area	Hydrology and Water Resources; Hydropower Management; Metaheuristic Algorithms
Type of data	Table and figures
How the data were acquired	Raw data were obtained by Meteorological and Hydrological Measurement and the data analyzed were obtained from the MATLAB software.
Data format	Raw and analyzed
Parameters for data collection	• Reservoir characteristic parameters (e.g., Minimum reservoir storages, Maximum reservoir storages, Power plant capacity (PPC), Annual potential energy production, Efficiency, Water release, and Downstream water level and so on); • The monthly time series of inflow, evaporation, precipitation, and release of the reservoir.
Description of data collection	Meteorological and Hydrological datasets are provided by the Khuzestan Water and Power Authority.
Data source location	The Karun-4 reservoir located in the Karun basin (50° 24′ E longitude, 31° 35′ N latitude), Southwest of Iran.
Data accessibility	All raw data and processed data are available in this data article as a supplementary file.

**Table:** 

**Value of the Data** • Data on the volumes of reservoir inflow, reservoir storage, evaporation from the reservoir, precipitation in the reservoir and release from the reservoir in the Karun-4 reservoir provide an overview of the operation of the reservoir between the years of 2010 and 2019. • These data can be used to analyze the water resources status and energy generation in the Karun-4 hydropower reservoirs operation. • The data will be useful for modeling purposes, especially relating to the Karun-4 reservoir operation. • They can also be used to examine the impact of Karun-4 reservoir operation on generating energy. • The analysis obtained herein with Evolutionary Algorithms (EAs) solver can serve as a standard benchmark for other researchers to compare their analysis of the other methods using this dataset. • Other researchers can use the MSA algorithm in solving large-scale problems such as the hydropower reservoir operation with confidently.

## 1 Data

**Fig. 1:**
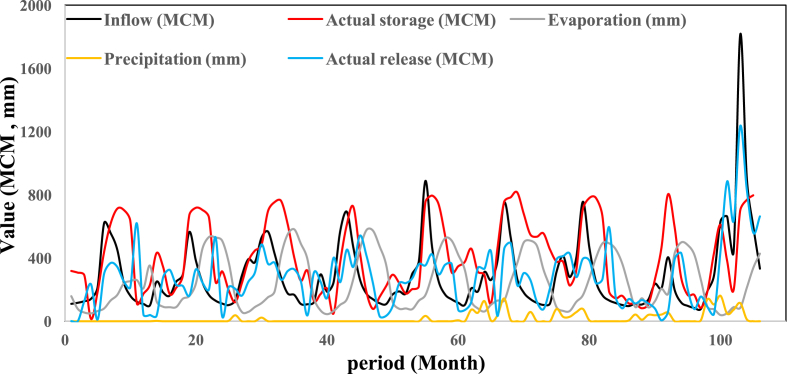
Time series chart of the dataset. The figure shows the time series meteorological and hydrological dataset consists of reservoir inflow, reservoir storage, evaporation from the reservoir, precipitation on the reservoir, and release of water through the power plant for a period of 106 months (from October 2010 to July 2019).

Water is a vital resource for socio-economic development in many parts of the world. Reservoir operation is an essential element in water resource planning and management. In the present study, Karun-4 hydropower reservoir operation is considered in terms of careful water demand management. The time series meteorological and hydrological dataset consists of reservoir inflow, reservoir storage, evaporation from the reservoir, precipitation on the reservoir, and release of water through the power plant for a period of 106 months (from October 2010 to July 2019). The utilized data are shown in Fig. 1. Reservoir inflow is the volume of water inflow to the Karun-4 reservoir, which is measured in million cubic meters (MCM). Reservoir storage is a volume of water storage of the Karun-4 reservoir at the beginning of each period, which is expressed in MCM. Evaporation from the reservoir is a depth of evaporation from the area of the Karun-4 reservoir at each period, which is expressed in millimeter (mm). Precipitation on the reservoir is a depth of precipitation in the area of the Karun-4 reservoir at each period, which is expressed in millimeter (mm). The release of water through the power plant is a volume of water outflow from the power plant of the Karun-4 reservoir at each period, which is expressed in MCM.

**Fig. 2:**
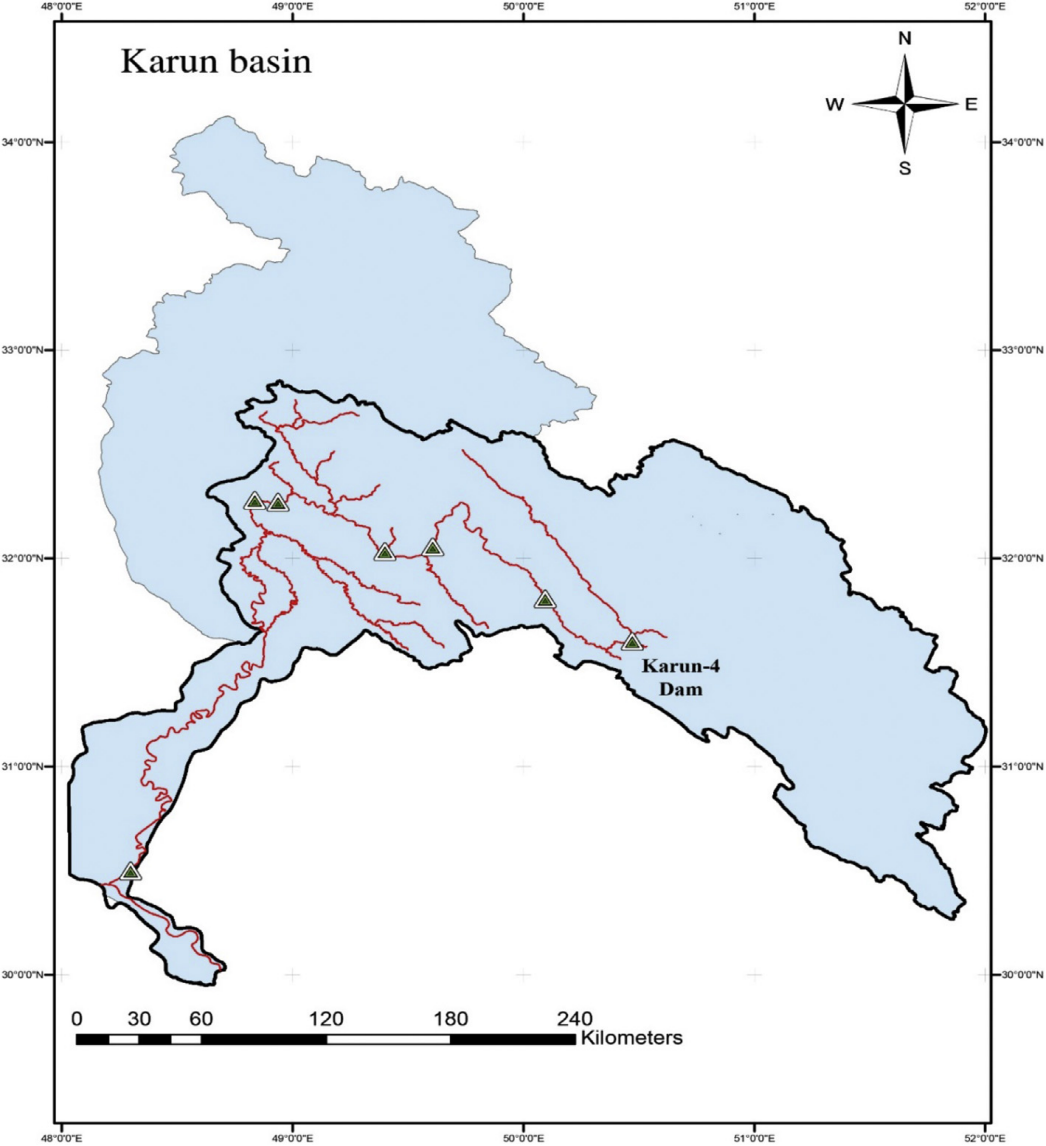
Location of the Karun-4 dam in the Karun basin (southwest of Iran).

**Fig. 3:**
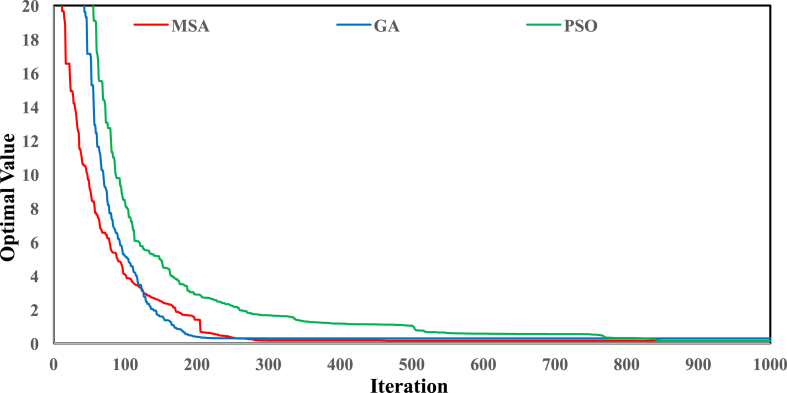
The convergence of applied algorithms in the Karun-4 hydropower reservoir. The figure shows the convergence rate of applied algorithms in reaching the optimum value for the hydropower operation problem. It also indicates the rapid convergence of the MSA in comparison with the other algorithms.

**Fig. 4:**
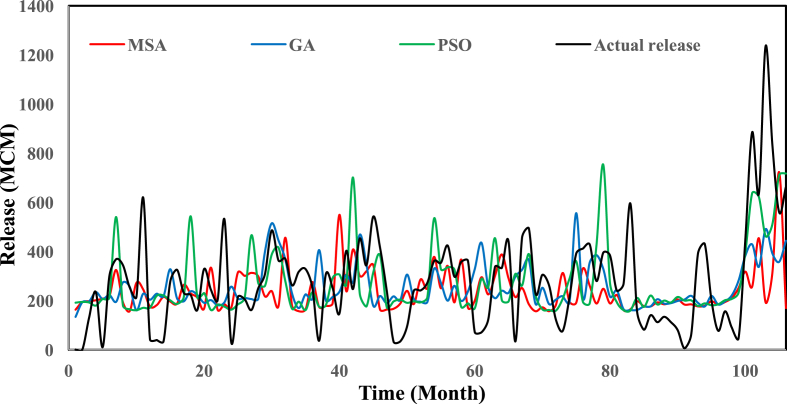
Water release patterns of applied algorithms in the Karun-4 hydropower reservoir. The figure shows the water release pattern for the operation of the Karun-4 hydropower reservoir using the MSA, GA, and PSO algorithms. The MSA algorithm was able to store and generate more energy by water releasing less for a period of 106 months. This indicates the high capability of the MSA in calculating near-optimal global solutions.

**Fig. 5:**
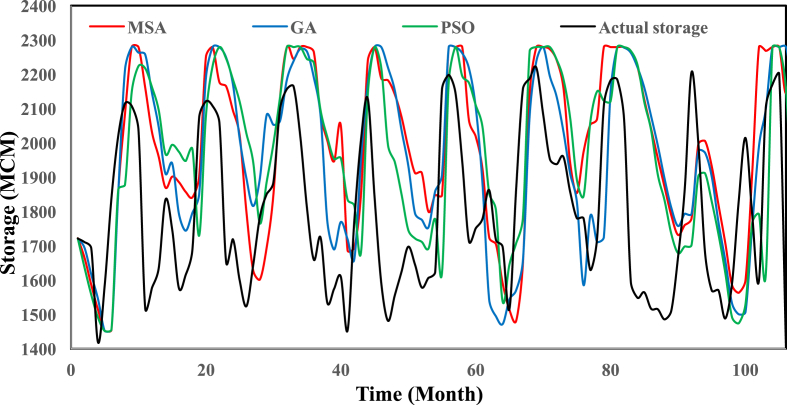
Water storage patterns of applied algorithms in the Karun-4 hydropower reservoir. The figure shows the water storage pattern for the operation of the Karun-4 hydropower reservoir using the MSA, GA, and PSO algorithms. According to this figure, the storage of the reservoir obtained by the runs of the investigated algorithms is better than the actual storage. Also, the figure shows the superior performance of the MSA algorithm compared to other algorithms.

Fig. 2 shows the location of the Karun-4 dam in the Karun basin. Table 1 gives the main characteristics of the Karun-4 dam reservoir. Table 2 displays the values of used algorithms parameters for the hydropower operation problem. Table 3 describes the objective value of objective functions and the average CPU run time obtained by each algorithm for the Karun-4 hydropower reservoir problem. Fig. 3 represents the convergence rate of applied algorithms in reaching the optimum value for 1000 iteration. Fig. 4 depicts the water release pattern for the operation of the Karun-4 hydropower reservoir for a period of 106 months (from October 2010 to July 2019). Finally, Fig. 5 shows the water storage pattern for the operation of the Karun-4 hydropower reservoir for this period.

**Table 1:** Main characteristics of the Karun-4 dam reservoir.

Parameters	Unit	Value
North latitude	Degree (°)	31° 35′
East longitude	Degree (°)	50° 24′
Minimum reservoir storages	MCM	1405
Maximum reservoir storages	MCM	2279
Power plant capacity (PPC)	MW	1000
Annual potential energy production	MWh	2107
Efficiency	Percent (%)	80

**Table 2:** Values of used algorithms parameters for hydropower operation problem.

MSA	parameter	iterations	Number of variables	Number of search agents	Number of Pathfinders	–
	Value	1000	106	100	20	–
GA	parameter	iterations	Number of variables	Number of genes	Mutation rate	Crossover rate
	Value	1000	106	100	0.01	0.8
PSO	parameter	iterations	Number of variables	Population Size	C_1_	C_2_
	Value	1000	106	100	1.49	1.49

**Table 3:** Analyses of 10 runs of the Karun-4 hydropower reservoir. The objective value of objective functions and the average CPU run time for each algorithm were presented in this table for the Karun-4 hydropower reservoir problem. Analysis of datasets in the table showed that MSA was able to produce superior solutions for the Karun-4 hydropower reservoir system.

Number of runs	MSA		PSO		GA	
	Optimal value	CPU time (s)	Optimal value	CPU time (s)	Optimal value	CPU time (s)
1	0.1559	21.82	0.1584	28.99	1.6918	48.71
2	0.1473	20.12	1.0708	30.35	1.4352	47.79
3	0.1470	21.46	0.2499	28.88	1.9616	40.88
4	0.1486	22.53	0.5463	28.93	1.4702	37.16
5	0.1508	21.58	0.2756	29.03	0.3762	48.23
6	0.1472	20.66	0.1704	29.01	0.6623	47.99
7	0.1506	19.7	0.2570	29.62	1.3717	47.93
8	0.1470	21.24	0.1591	29.6	0.9225	48.09
9	0.1473	20.42	0.732	29.17	0.5495	47.14
10	0.1471	21.76	0.1823	28.93	0.3026	47.62

Best	**0.1470**		0.1584		0.3026	
Worst	**0.1559**		1.0708		1.9616	
Average	**0.1489**		0.3802		1.0744	
SD	**0.0029**		0.3078		0.5864	
Coefficient of variation	**0.0192**		0.8096		0.5458	
Best CPU time (s)	**19.7**		28.88		37.16

## 2 Experimental design, materials and methods

In this data article, using the time-series dataset, a model based on Moth Swarm Algorithm (MSA) was developed for optimal hydropower operation of the Karun-4 Reservoir. The details of the MSA algorithm were provided by Mohamed et al. (2017) [1]. The MSA algorithm was compared with other well-known developed evolutionary algorithms, including GA and PSO algorithms [[2], [3], [4]]. It is noteworthy that all the studied metaheuristic algorithms were coded in MATLAB software.

### 2.1 Experimental design

The simulation optimization model for producing a time-series dataset of the highest amount of energy of the Karun4 reservoir was structured in a monthly time step during the period 2010–2011 to 2018–2019. Objective functions and constraints of the Karun-4 reservoir are as follows:

(1) $$MinF=\sum_{t=1}^{T}\left(1-\frac{P_{t}}{PPC}\right)$$

(2) $$P_{t}=g\times e_{t}\times \left(\frac{RP_{t}}{PF}/Mul_{t}\right)\times \left(\bar{H_{t}}-TW_{t}\right)/1000$$

(3) $$\bar{H_{t}}=\left(H_{t}+H_{t+1}\right)/2$$

(4) $$H_{t}=a_{0}+a_{1}.S_{t}+a_{2}.S_{t}^{2}+a_{3}.S_{t}^{3}$$

(5) TWt=b0+b1.RetPower+b2.(RetPower)2+b3.(RetPower)3

(6) RPSt=RetPower−RPt

(7) $$0\leq P_{t}\leq PPC$$

(8) St+1=St+Qt−RetPower−Spt−Losst

(9) $$Loss_{t}=\left(Ev_{t}-R_{t}\right)\times \bar{A_{t}}/1000$$

(10) $$\bar{A_{t}}=\left(A_{t}+A_{t+1}\right)/2$$

(11) $$A_{t}=c_{0}+c_{1}.S_{t}+c_{2}.S_{t}^{2}+c_{3}.S_{t}^{3}$$

(12) $$S_{\min}\leq S_{t}\leq S_{\max}$$

where $P_{t}$ is the electricity produced by the power plant (MW), PPC is the total power plant capacity (MW), T is the total number of hydropower operation periods of the Karun-4 reservoir. In addition, g is gravitational acceleration, $e_{t}$ is efficiency of the Power plant, PF is the plant factor, $RP_{t}$ is the water release through the power plant to generate power (MCM) in period t, $Mul_{t}$ is conversion factor from million cubic meters to cubic meters per second during period t, $H_{t}$ and $H_{t+1}$ are reservoir water level at the beginning and end of period t (m), respectively, $TW_{t}$ is reservoir tail-water level, which is assumed constant for all periods during period t (m), RetPower is water release through the power plant (MCM) in period t, $RPS_{t}$ is the overflow volume through the power plant in period t (MCM), $S_{t}$ is the reservoir storage (MCM), $Q_{t}$ is the reservoir inflow (MCM), $Sp_{t}$ is the spill overflow from the reservoir during period t (MCM), $Loss_{t}$ is the loss from reservoir (MCM), $Ev_{t}$ is the depth of evaporation from the reservoir (m), $R_{t}$ is the depth of precipitation on the reservoir (m), $A_{t}$ and $A_{t+1}$ are area of the reservoir lake at the beginning and end of period t (Km^2^), respectively, $S_{\min}$ is the minimum storage (MCM), $S_{\max}$ is the maximum storage capacity (MCM), and $a_{i}$, $b_{i}$, and $c_{i}$ are the coefficients of the Storage-Area-Depth relationships for the reservoir.

### 2.2 Analysis of datasets

The analyses of this data article showed that the best solution achieved by the MSA, GA, and PSO algorithms for the Karun-4 hydropower reservoir problem were 0.147, 0.3026, and 0.1584, respectively. The analyses revealed that the MSA algorithm was the superior algorithm in the optimal operation of the Karun-4 hydropower reservoir.

All analyses of this research for each algorithm are presented in Table 3 and Fig. 3, Fig. 4, Fig. 5.

## Data availability statement

All datasets, models, or codes generated or used during the article are available from the corresponding author by request.
